# La version française de la liste de vérification CRISP : un outil pratique pour la publication de la recherche en soins primaires en français

**DOI:** 10.4102/phcfm.v18i1.5398

**Published:** 2026-06-18

**Authors:** Jean-Pierre Fina-Lubaki, Williams R. Phillips, Klaus B. von Pressentin

**Affiliations:** 1 Département de médecine de famille et soins de santé primaires, Faculté de Médecine, Université Protestante au Congo, Kinshasa, Democratic Republic of the Congo; 2 Department of Family Medicine and Primary Care, School of Clinical Medicine, Faculty of Health Sciences, University of the Witwatersrand, Johannesburg, South Africa; 3 Department of Family Medicine, University of Washington, Seattle, United States of America; 4 Department of Family, Community and Emergency Care, Faculty of Health Sciences, University of Cape Town, Cape Town, South Africa

## Abstract

**The French version of the CRISP checklist: A practical tool for publishing primary care research in French.** This editorial introduces the French version of the Consensus Reporting Items for Studies in Primary Care (CRISP) checklist, a reporting tool developed to improve the quality, relevance, and transparency of primary care publications. It notes that primary care research has unique methods, populations, and health system contexts, and thus needs tailored reporting guidance. The article describes the checklist’s development via international consensus and its flexibility for quantitative, qualitative, and mixed-methods research. The checklist assists authors, reviewers, editors, teachers, and study planners. The editorial stresses the importance of a French version, given French’s global presence, especially in Africa, while English dominates scientific publishing. This language barrier hinders French-speaking researchers. Offering a French version improves access and supports clearer communication of research in Francophone and African settings. The editorial concludes that the French CRISP checklist strengthens reporting and helps to interpret and apply primary care research findings.

Les soins primaires font référence aux soins fournis aux populations au point d’entrée des systèmes de santé.^1^ Ils concernent tous les membres de la population sans distinction. Depuis des décennies, l’importance des soins de santé primaires pour garantir un accès équitable aux services de santé et améliorer la santé de la population est bien établie. De nombreuses stratégies de santé reposent donc sur des soins de santé primaires de haute qualité.

À la première ligne des soins, les problèmes de santé se présentent à un stade indifférencié et se manifestent par des symptômes vagues ou non spécifiques nécessitant une approche experte.^2^ La recherche en soins primaires est cruciale et devrait être encouragée, car elle aide à mieux définir les besoins de la population et à mettre en évidence les spécificités liées à leurs valeurs, cultures et aux considérations dans le développement des stratégies de santé.^3,4^ La recherche en soins primaires a ses propres spécificités, notamment en ce qui concerne les sujets abordés, les méthodologies utilisées et surtout la communication des résultats de recherche.

De plus en plus, une recherche de haute qualité va de pair avec des publications qui respectent les recommandations résumées dans les listes de vérification pour la communication des résultats de recherche. Pour répondre aux besoins de la recherche en soins primaires, le groupe de travail sur les éléments de rapport d’études en soins primaires établis par consensus (ÉRÉSPC ou *Consensus Reporting Items for Studies in Primary Care* [CRISP], en anglais) a mené des recherches rigoureuses pendant plus de cinq ans, impliquant 596 personnes provenant de plus de 29 pays. Ce travail novateur a abouti à la publication de la liste de vérification des ÉRÉSPC en 2023.^5^ Les membres de la communauté des soins primaires ont sélectionné 24 éléments jugés essentiels pour que les rapports de recherche soient valides et applicables dans les contextes et les populations de patients des soins primaires. Les 24 éléments de la liste de vérification des ÉRÉSPC décrivent l’équipe de recherche, les patients, les participants à l’étude, les conditions de santé, les consultations cliniques, les équipes de soins, les interventions, les mesures de l’étude, les lieux de soins et la mise en œuvre des résultats dans les soins primaires.^5^

L’un des principes fondamentaux de la liste de vérification des ÉRÉSPC est qu’il reflète les bases des soins centrés sur le patient, orientés vers la communauté et axés sur les problèmes.

La liste de vérification des ÉRÉSPC est suffisamment flexible pour s’adapter à la variété des méthodes de recherche, des types d’études, des sujets et des systèmes de santé à la première ligne des soins. La liste de vérification des ÉRÉSPC est aussi bien adaptée pour la recherche quantitative, qualitative et mixte; en outre, elle inclut une description détaillée du contexte, ce qui est très important dans la recherche qualitative. Il convient de noter que tous les éléments ne s’appliquent pas à toutes les études.^5,6^ Aucune restriction n’est imposée en matière de reportage efficace et créatif. La liste de vérification est soutenue par l’énoncé des ÉRÉSPC et un guide sur son utilisation, avec des explications et des exemples. Tous les documents et traductions des ÉRÉSPC sont disponibles sur le site web CRISP : https://crisp-pc.org.

La liste de vérification offre un outil pour les auteurs, les réviseurs et les éditeurs. Les ÉRÉSPC ont été conçues pour améliorer la communication des résultats de la recherche en soins primaires, mais elles sont également utiles pour la révision des manuscrits. La liste de vérification des ÉRÉSPC peut également soutenir la planification des études et l’enseignement des méthodes de recherche. Il est important de noter que la liste de vérification des ÉRÉSPC s’applique à toute la recherche en soins primaires – non seulement aux recherches conduites par des chercheurs en soins primaires, mais également aux études réalisées par d’autres chercheurs dans les milieux de soins primaires.^7^

La liste de vérification des ÉRÉSPC est enregistrée sur le réseau EQUATOR et disponible en plusieurs langues. Une version française a été développée^8^ et est disponible sur le site Web de *Canadian Family Physician* dans la table des matières du numéro de janvier 2025 à la page e19.

Le français est la cinquième langue la plus parlée au monde, avec environ 380 millions de locuteurs. Il est parlé sur les cinq continents et gagne en popularité, particulièrement en Afrique. Dans le monde scientifique, il y a une tendance à privilégier la publication en anglais pour un meilleur impact.^9,10^ Néanmoins, traduire des articles scientifiques du français vers l’anglais présente de nombreux défis.^11^ Une étude a mentionné les défis suivants lors de la traduction de documents du français vers l’anglais : compétence linguistique limitée dans la langue source et la langue cible, technicité des documents, polysémie et faux amis, mots liés à la culture, et temps insuffisant pour se concentrer sur le travail de traduction. En effet, Le français possède des caractéristiques culturelles, idiomatiques et stylistiques qui ne se traduisent pas directement en anglais.^12^ Tous ces défis pourraient conduire à déformer le sens des mots.^13^

La disponibilité de la liste de vérification des ÉRÉSPC en français représente donc un avantage considérable pour les chercheurs en soins de santé primaires dont la langue première ou officielle est le français. En raison de son insistance sur des descriptions détaillées des contextes et des systèmes de santé dans lesquels la recherche est menée, elle est particulièrement utile dans les contextes africains. Les auteurs africains peuvent donc utiliser la liste de vérification des ÉRÉSPC pour aider les lecteurs à mieux comprendre, interpréter et appliquer les résultats de leurs recherches.

Les revues *African Journal of Primary Health Care and Family Medicine* et *Canadian Family Physician*, qui publient en français, encouragent les auteurs à utiliser la liste de vérification des ÉRÉSPC.^7,8^ La liste de vérification des ÉRÉSPC est approuvée par le comité exécutif de la *World Organization of Family Doctors* (WONCA) depuis août 2023, ainsi que par le *North American Primary Care Research Group* (NAPCRG).^5^

Le groupe de travail sur la liste de vérification des ÉRÉSPC s’engage à une évaluation et à des mises à jour continues, en accueillant les suggestions des auteurs. Il souligne que la plupart des publications devraient suivre deux lignes directrices : la liste de vérification des ÉRÉSPC pour la recherche en soins primaires et la ligne directrice EQUATOR pertinente pour le type d’étude.^5^

## Déclaration

Le premier auteur, Jean-Pierre Fina-Lubaki, sert en tant qu’assistant éditeur de cette revue. Jean-Pierre Fina-Lubaki n’a aucun autre conflit d’intérêt à déclarer.
